# Serum Folate Related to Five Measurements of Obesity and High-Sensitivity C-Reactive Protein in Korean Adults

**DOI:** 10.3390/nu14173461

**Published:** 2022-08-24

**Authors:** Mee-Ri Lee, Sung Min Jung

**Affiliations:** 1 Department of Preventive Medicine, Soonchunhyang University College of Medicine, Cheonan-si 31151, Korea; 2 Department of Surgery, Inje University, Ilsan Paik Hospital, Goyang-si 10380, Korea

**Keywords:** folic acid, body mass index, waist–height ratio, waist circumference, a body shape index, body roundness index

## Abstract

This study investigated the effects of folic acid on obesity and high-sensitivity C-reactive protein (CRP) levels. Using data from the Korea National Health and Nutrition Examination Survey (KNHANES VII 2016–2018), 6394 adults (aged 19–80 years) who met the study criteria were identified and divided into young, middle-aged, and older adult groups. The analysis assessed associations using logistic regression for complex samples. Obesity was measured using body mass index (BMI), waist circumference (WC), waist-to-height ratio (WHtR), a body shape index (ABSI), and body roundness index (BRI). The odds ratio (OR) of obesity based on BMI were statistically significant for young adults and older participants with low levels of folic acid compared to those with high levels (OR: 1.33 and 1.58, respectively). The OR of obesity based on BMI, WC, WHtR, ABSI, and BRI was significant with low levels of folic acid in middle-aged individuals (OR: 1.36, 1.32, 1.41, 1.29, and 1.47, respectively). Low folate levels were related to higher high-sensitivity CRP levels in middle-aged patients. In conclusion, folate level showed a significant inverse association with obesity and inflammatory biomarkers, especially in the middle-aged group. Further longitudinal or randomized controlled trials are needed to confirm and expand our results.

## 1. Introduction

Obesity and being overweight are among the most important health burdens worldwide [1]. Global obesity has nearly tripled since 1975, with 39% of adults being overweight and 13% being obese in 2016 [2]. Obesity affects insulin resistance [3], cardiovascular diseases [4], ischemic stroke [5], cancers [6], and increases mortality.

Although high-carbohydrate and high-fat diets are associated with obesity [7], association between micronutrient status and obesity is unclear. A previous study revealed that vitamin B12 is inversely associated with obesity [8] and vitamin D supplementation in obese subjects could affect weight loss and decrease fat mass [9].

Folate is an essential water-soluble vitamin that is present in fruits, green leafy vegetables, and the liver [10] It reduces the risk of serous neural tube defects in babies, and some countries have mandatory folic acid fortification of flour [11].

Previous studies showed that lower folate associated with obesity [12,13]. However, a review article reported that there was no relationship between folate concentration and body mass index (BMI) [14]. A cross-sectional study showed no association between BMI and waist circumference (WC) [15]. Inconsistent evidence has revealed that folate levels may be associated with obesity.

BMI is a useful population-based measurement of general obesity and the well-known World Health Organization (WHO) categories [16,17]. However, BMI has limitations; thus, WC and waist-to-height ratio (WHtR) are preferred alternatives [18].

Two novel obesity indices were introduced. Krakauer and Krakauer developed a body shape index (ABSI) that correlates with abdominal adiposity deposition in 2012 [19]. In 2013, Thomas et al. (2013) developed the body roundness index (BRI) as a predictor of visceral adiposity tissue and body fat percentage [20]. In obesity, adipocytes are enlarged and the secretion of inflammatory factors such as high-sensitivity C-reactive protein (hs-CRP) is increased [21]. Cross-sectional paper for Asia found that central obesity was associated with hs-CRP [22,23]. Some studies showed subjects with a high BMI and the health metabolic profile was associated with increased hs-CRP compared to non-obese subjects [24]. However, another study found that hs-CRP was not related to future weight gain or increased waist circumference in Finnish adults [25].

Despite some studies on the association folic acid and obesity, to our knowledge, no study has used the ABSI and BRI. Few studies have used all five obesity measures. Therefore, we investigated the association between folic acid and five obesity measurements (BMI, WC, WHtR, ABSI, and BRI) and inflammatory biomarkers (hs-CRP) in Korean adults using data from the Korea National Health and Nutrition Examination Survey (KNHANES VII 2016–2018).

## 2. Materials and Methods

### 2.1. Study Design and Study Population

**Figure 1:**
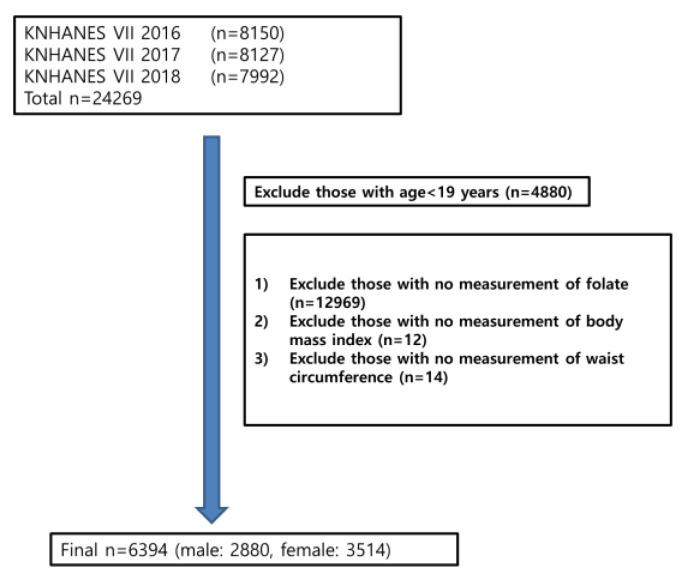
Flow chart showing exclusion procedure of participants.

The KNHANES is a study that is representative of the Korean population and used a complex study design to randomly select subjects to collect data. It is an annual study conducted by the Ministry of Health and Welfare. The flow chart was shown in Figure 1 and 6394 adults were included in the analysis. Subjects were divided into young adult (19–39 years old), middle-aged (40–64 years old), and older adult (65–80 years old) age groups.

### 2.2. Measurement of Folic Acid and High-Sensitivity C-Reactive Protein

Folate was detected in the samples using ARCHITECT i4000Sr (Abbott Laboratories, Abbott Park, IL, USA) and ARCHITECT Folate-only reagent with chemiluminescent microparticle immunoassay methods.

Hs-CRP was measured using Cobas immunoturbidimetry (Roche, Berlin, Germany).

### 2.3. Obesity Assessment

BMI was calculated as weight divided by height squared (kg/m^2^). A high BMI was defined as 25 kg/m^2^ or higher. High WC was defined as WC ≥ 85 cm in women and ≥90 cm in men. WHtR was measured as waist circumference divided by height. A high WHtR was defined as 0.5 and higher. The calculations of ABSI and BRI are described below (WC (m), height (m), and BMI (kg/m^2^)) [19].

$$\mathrm{ABSI}\;=\frac{1000*WC}{BMI^{2/3}\;\;Height^{1/2}}\;$$

$$\mathrm{BRI}=364.2-365.5\;*\;\sqrt{1-(\frac{(WC/{\left(2\pi \right)}^{2}}{{\left(0.5height\right)}^{2}}})$$

High ABSI was defined as a mean or higher in each age group (means of total subject, young adult, middle-aged, and older adults were 77.56, 74.94, 77.56, and 81.62, respectively).

High BRI was defined as a mean or higher in each age group (means of total subject, young adult, middle-aged, and older adults were 3.49, 2.94, 3.53, and 4.24, respectively).

### 2.4. Covariates

Education was defined as the period of education divided into three categories: less than 9 years, 10–12 years, and more than 12 years. Smoking status was divided into three groups: lifetime non-smokers, former smokers (those who had smoked in the past but not currently), and current smokers (those who had smoked more than 100 cigarettes in their lifetime and were still smoking). Drinkers were defined as those who drank at least one drink per month in the past year. Physical activity was defined as at least 150 min per week of moderate-intensity, 75 min or more per week of high-intensity, or a combination of moderate-intensity and high-intensity physical activity. All covariates were information obtained from the subject’s self-reported questionnaire.

### 2.5. Statistical Analysis

We analyzed KNHANES VII (2016–2018) data using complex sample analysis, according to the statistical guidelines provided by the Centers for Disease Control and Prevention.

Folic acid levels were divided into two groups according to the mean. The mean folic acid levels for the total subjects, young adults, middle-aged adults, and older adults were 7.39, 6.49, 7.76, and 7.91, respectively).

One-way ANOVA or chi-square test was used to compare differences in sociodemographic and clinical characteristics according to the three age groups, and the Student’s *t*-test or chi-square test was used for comparison according to the folate level.

Pearson’s correlation coefficient was used to test correlations between BMI and serum folate, and since folate did not have a normal distribution, it was analyzed by converting it to normal by log transform.

Odds ratios (OR) were calculated by dividing the odds of the occurrence of high BMI, WC, WHtR, ABSI, BRI, and hs-CRP (in those with lower-than-average folic acid levels) by the odds of the occurrence of high BMI, WC, WHtR, ABSI, BRI, and hs-CRP (in those with above-average folic acid levels). Multivariate logistic analysis was adjusted for age, sex, education, smoking status, alcohol consumption, and physical activity.

*p*-value of 0.05 or less is defined as statistically significant. All statistical analyses and graphs were performed using Stata version 17 (Stata Corp., College Station, TX, USA) and the R software, version 4.0.2 (The Comprehensive R Archive Network: http://cran.r-project.org (accessed on 13 April 2022).

## 3. Results

A total of 6394 participants (mean 49.3 years, range (19–80)) were included in the data, including young adults (2013, mean 30.4 years), middle-aged adults (3086, mean 51.9 years), and older adults (1295, 72.5 years old). The mean of folic acid for the subjects, young adults, middle aged, and the older adults, was 7.39 ng/mL, 6.49 ng/mL, 7.76 ng/mL, and 7.91 ng/mL, respectively).

Table 1 shows the differences in sex, education, smoking status, alcohol consumption, physical activity, BMI, WC, WHtR, ABSI, BRI, hs-CRP, and folic acid among the demographic and anthropometric characteristics of the three population groups.

Table 2 compares the variables between high- and low-serum folate level groups in young adults, middle-aged, and older adults. In young adults, the high-folate group had the following characteristics when compared to the low-folate group: women (70.9% vs. 43.3%), high education (84.8% vs. 80.6%), non-smoker (68.8% vs. 54.9%), non-drinker (36.8% vs. 30.0%), and lower level of BMI, WC, WHtR, ABSI, and BRI. In middle-aged adults, the high-folate group compared with the low-folate group showed more women (71.7% vs. 44.4%), low education (24.1% vs. 20.0%), non-smoker (72.7% vs. 48.2%), non-drinker (49.6% vs. 38.0%), physical activity (49.8% vs. 39.9%), and a lower level of BMI, WC, WHtR, ABSI, BRI, and hs-CRP. In older adults, the high-folate group was more women (63.8% vs. 45.6%), non-smoker (70.2% vs. 51.2%), non-drinker (70.3% vs. 59.8%), physical activity (37.9% vs. 29.9%), and lower level of WC and ABSI compared to the low-folate group.

Using Pearson’s correlation coefficient, significant negative correlation was found between log transform of folate and BMI in young adults and middle-aged adults. The scatter plot is presented in Supplementary Figure S1.

**Figure 2:**
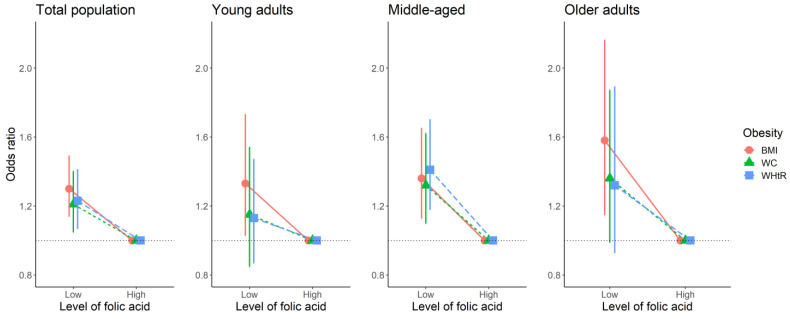
The association between binary of folic acid and BMI, WC, WHtR, and ABSI in each age group using multivariate logistic regression. Abbreviations: BMI, body mass index; WC, waist circumference; WHtR, waist-to-height ratio.

**Figure 3:**
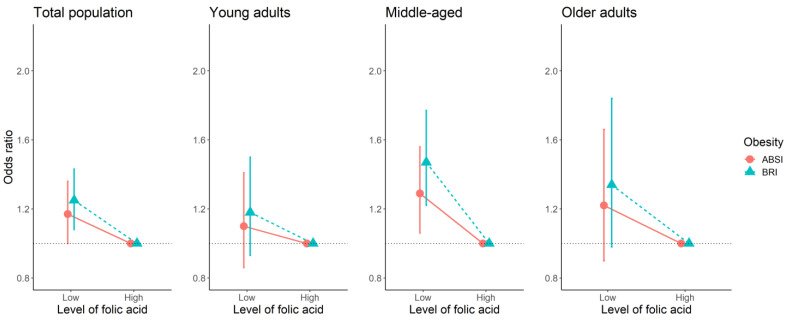
The association between the binary of folic acid and new obesity indices (ABSI, BRI) in each age group using multivariate logistic regression. Abbreviations: ABSI, a body shape index; BRI, body roundness index.

After adjustment for all covariates, among participants who had low levels of folic acid, the ORs of having high BMI, WC, WHtR, ABSI, and BRI were 1.30 (95% confidence interval (CI): 1.14–1.49), 1.21 (95% CI: 1.05–1.40), 1.23 (95% CI: 1.07–1.41), 1.17 (95% CI: 1.00-–1.36), and 1.25 (95%CI: 1.08–1.43) compared to those with high levels in all participants (Figure 2 and Figure 3). In middle-aged adults, lower folic acid levels had significant ORs of five obesity measurements (BMI, WC, WHtR, ABSI, and BRI) compared with high folic acid levels (OR (95% CI): 1.36 (1.13–1.65); 1.32 (1.1–1.62); 1.41 (1.18–1.7); 1.29 (1.06–1.56); 1.47 (1.22–1.77). Lower folate levels were related to high BMI in young adults and the older adults (OR (95% CI): 1.33 (1.03–1.73) and 1.58 (1.15–2.16), respectively), but not with other obesity variables (Figure 2 and Figure 3).

**Figure 4:**
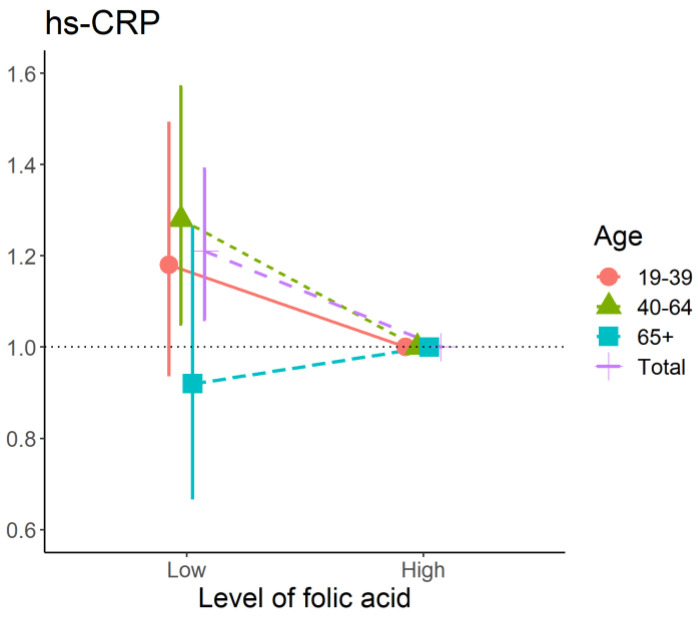
The association between the binary of folic acid and high–sensitivity C-reactive protein in each age group using multivariate logistic regression. Abbreviations: hs-CRP, high-sensitivity C-reactive protein.

In middle-aged adults, lower levels of folate were significantly related with hs-CRP (OR (95%CI): 1.28 (1.05–1.57)), but not in the young adults or the older adults using multivariate logistic regression (Figure 4).

## 4. Discussion

This study examined the association of serum folate levels with all five measures of obesity (BMI, WC, WHtR, ABSI, and BRI) and hs-CRP levels in a nationally representative sample of Korean adults, especially middle-aged adults. However, in young adults and older adults, only obesity measured by BMI was statistically significant. In this study, the level of folic acid increased with age, which is similar to the results of previous studies that used representative US data [26].

Our findings are similar to those of a previous study. A study of 2695 Brazilian subjects showed that dietary folate intake was negatively associated with overweight (BMI ≥ 25 kg/m^2^) and obese (BMI ≥ 30 kg/m^2^) prevalence [27].

A case–control study of 421 healthy participants (aged 20–40) revealed that lower folate serum concentrations were negatively associated with BMI (≥ 25 kg/m^2^), waist-to-hip ratio (WHR), WC, and fat percentage [12]. Recent Korean study reported that serum folate levels were associated with WC (≥85 cm) in 1730 premenopausal women [13]. In the meta-analysis of a randomized controlled trial, folic acid supplementation significantly reduced serum levels of hs-CRP [28]. Dose–response analysis demonstrated a significant association between an elevated dosage of folic acid supplementation and lower CRP concentrations [29].

BMI, WC, WHtR, ABSI, and BRI have different aspects for obesity measurement. BMI refers to “total obesity” but did not account for an individual’s body composition and body fat distribution [30]. WC reflects abdominal obesity using an absolute measure and does not indicate whether fat is concentrated in the abdomen or spread throughout the body [31]. Although WC increases according to the body size, it can be classified as obese if a normal-weighted person has an extremely large waist [32]. Some studies used WHtR to adequately capture the distribution of body fat for assessing obesity and body composition and found that WHtR was a better predictive indicator of cardiovascular disease than other common indices of obesity (BMI, WHR, and WC) [33,34]. In a study in England, waist circumference indices (WHtR, WC) were consistently superior to BMI when explaining or predicting cardiometabolic risk (high-density lipoprotein cholesterol, glycated hemoglobin, systolic and diastolic blood pressure) [18]. ABSI was developed to compensate for the BMI and WC limitation and was obtained from allometric regression and designed to be minimally associated with weight, height, and BMI [31,32]. The BRI was used to predict the percentage of body fat and visceral fat, which are not clearly addressed by BMI [20].

The association of folic acid with all measures of obesity and hs-CRP, especially in middle-aged individuals, may be due to age-related changes in body composition. Previous studies explain that muscle mass increases until the age of 40, but when people reach the middle age of 40 or more, their skeletal muscle gradually decreases, fat increases and relocation occurs, and sarcopenic obesity occurs at an old age [35]. In addition, the measurement of BMI is more likely to include people with a lot of muscle mass as obese in the younger group, and people with very little muscle mass and a lot of fat in the older adults are more likely to be judged as normal.

Several possible mechanisms may underlie the inverse association between serum folate levels and obesity and hs-CRP levels, but these are still not fully understood. One possible explanation is homocysteine. Absorbed folate is metabolized to 5-methyltetrahydrofolate (5-methylTHF), which supplies a methyl group that converts homocysteine (Hcy) to methionine [36]. Folate deficiency induces the development of hyperhomocysteinemia [37], which is associated with suppressed lipolysis in adipocytes and adipose tissues [38]. Hcy also stimulates the expression of inflammatory cytokines by enhancing poly adenosine diphosphate (ADP) ribose polymerase activation and prompting nuclear factor kappa B (NF-kB) activation [29]. In vivo and in vitro studies have demonstrated that folate deficiency increases fat accumulation and leptin production in adipocytes, which may contribute to the increase in obesity prevalence and inflammatory disease [39]. Another explanation is that obesity is regarded as chronic low-grade inflammation with permanently increased oxidative stress [40]. A double-blind placebo-controlled study showed a reduction in oxidative stress after folate administration in 30 healthy postmenopausal Caucasian women [41].

US centers for disease control and prevention recommendations require that all women of the childbearing age consume 400 micrograms (mcg) of folic acid daily to prevent neural tube defects [42]. Typical dosages for folic acid supplementation in the United States range from 400 to 800 mcg folic acid for adults and 200 to 400 mcg folic acid for children’s multivitamins [43]. A study using the US data found that about one-third of men and women of all ages used folic acid supplements, and the average level of non-supplement users was 11% lower than overall [44]. They also found that more than half of women who did not take folic acid supplements consumed less folic acid than the WHO recommended [44].

This study has several important advantages. This is the first study to investigate serum folate levels related to ABSI and BRI using data representing the entire Korean population. In addition, the results were shown in three age groups: young adults, middle-aged adults, and older adults, rather than in a limited age group. Finally, based on recent cohort study for Chinese adults showing that BMI and hs-CRP were jointly related to metabolic health [45], our findings suggest that raising folic acid concentrations may be beneficial for metabolic health as well as obesity.

However, this study also has some limitations. Firstly, because of the cross-sectional nature of the study, the reverse relationship was not excluded; thus, it does not allow inferences about causality. Another study showed that obese participants had lower serum folate levels because of folate metabolic changes, reduced supplement use, and unhealthy diets [46]. Secondly, these data are only for Korean adults, so it may be difficult for other races and nationalities. Thirdly, since the KNHANES data do not have information about the folic acid supplements taken by participants, it is not possible to examine the effects of folic acid supplementation on obesity.

## 5. Conclusions

In middle-aged individuals, higher folate levels may improve obesity and inflammation. Future longitudinal or randomized controlled trials for the appropriate management of middle-aged individuals with lower folate levels are needed to confirm and expand our findings.

## Figures and Tables

**Table 1:** Participants’ sociodemographic and clinical characteristics according to age group.

Variable	Total	19–39	40–64	65+	*p* Value
n	6394	2013	3086	1295	
Age, y	49.3 ± 16.3	30.4 ± 6.1	51.9 ± 7.0	72.5 ± 5.1	<0.001
Women	3514(50.3)	1092(47.4)	1733(51.0)	689(54.1)	0.004
Education					
Low	1496(20.0)	23(0.8)	639(18.8)	834(65.3)	<0.001
Medium	1680(26.9)	320(15.6)	1113(37.7)	247(20.3)	
High	2923(53.1)	1589(83.6)	1189(43.5)	145(14.4)	
No response	295	81	145	69	
Smoking status					
Non-smoker	3752(57.7)	1203(58.9)	1798(55.8)	751(60.6)	<0.001
Former smoker	1339(21.9)	314(17.0)	632(22.6)	393(30.0)	
Current smoker	1230(20.4)	476(24.1)	630(21.6)	124(9.4)	
No response	73	20	26	27	
Alcohol consumption					
Non-drinker	2788(40.1)	654(31.2)	1317(40.4)	817(64.3)	<0.001
Alcohol drinker	3545(58.9)	1346(68.8)	1744(59.6)	455(35.7)	
No response	61	13	25	23	
Physical activity					
No	3336(52.3)	877(43.1)	1644(54.4)	815(66.1)	<0.001
Yes	2762(47.7)	1054(56.9)	1301(45.6)	407(33.9)	
No response	296	82	141	73	
BMI					
<25	4195(65.5)	1412(69.5)	1974(65.5)	809(62.6)	0.0002
≥25	2199(34.5)	601(30.5)	1112(36.5)	486(37.4)	
WC					
Male < 90, Female < 85	4556(72.5)	1577(79.0)	2224(72.0)	755(60.1)	<0.001
Male ≥ 90, Female ≥ 85	1838(27.5)	436(21.1)	862(28.0)	540(39.9)	
WHtR					
WHtR < 0.5	3147(52.1)	1409(71.0)	1433(47.5)	305(24.7)	<0.001
WHtR ≥ 0.5	3247(47.9)	604(29.0)	1653(52.5)	990(75.3)	
ABSI	77.56(4.64)	74.94(3.96)	77.56(3.92)	81.62(4.25)	<0.001
BRI	3.49(1.14)	2.94(1.13)	3.53(1.06)	4.24(1.27)	<0.001
hs-CRP, mg/L	1.17 ± 2.00	1.10 ± 1.96	1.09 ± 1.70	1.49 ± 2.60	<0.001
Folic acid, ng/mL	7.39 ± 3.58	6.49 ± 3.40	7.76 ± 3.45	7.91 ± 3.88	<0.001

Data were presented as mean ± standard deviation or number (percentage). χ^2^ test and one-way ANOVA were used for categorical and continuous variables, respectively. Bold numbers highlight the statistical significance. Abbreviations: ABSI, a body shape index; BMI, body mass index; BRI, body roundness index; hs-CRP, high-sensitivity C-reactive protein; SD, standard deviation; WC, waist circumference; WHtR, waist-to-height ratio.

**Table 2:** Participants’ sociodemographic and clinical characteristics according to the level of serum folate.

	Total			19–39			40–64			65+		
	Low	High	*p*	Low	High	*p*	Low	High	*p*	Low	High	*p*
Age, y	46.76 ± 16.98	51.78 ± 15.19	**<0.001**	29.75 ± 6.26	31.51 ± 5.63	**<0.001**	51.27 ± 6.98	52.83 ± 6.84	**<0.001**	72.82 ± 5.11	72.16 ± 4.99	**0.021**
women	1296 (41.75)	2218 (67.42)	**<0.001**	527 (43.3)	565 (70.9)	**<0.001**	778 (44.4)	955 (71.7)	**<0.001**	345 (45.6)	344 (63.8)	**<0.001**
Education												
Low	645 (22.0)	851 (26.9)	**<0.001**	18 (1.5)	5 (0.6)	**0.027**	333 (20.0)	306 (24.1)	**0.020**	495 (70.5)	339 (64.7)	0.092
Medium	787 (26.8)	893 (28.2)		208 (17.9)	112 (14.6)		656 (39.3)	457 (35.9)		132 (18.8)	115 (21.9)	
High	1505 (51.2)	1418 (44.9)		937 (80.6)	652 (84.8)		680 (40.7)	509 (40.0)		75 (10.7)	70 (13.4)	
No response	167	128		53	28		85	60		54	15	
Smoking status												
Non-smoker	1502 (49.0)	2250 (69.2)	**<0.001**	662 (54.9)	541 (68.8)	**<0.001**	838 (48.2)	960 (72.7)	**<0.001**	376 (51.2)	375 (70.2)	**<0.001**
Former smoker	685 (22.3)	654 (20.1)		173 (14.3)	141 (18.0)		408 (23.5)	224 (16.9)		258 (35.2)	135 (25.3)	
Current smoker	880 (28.7)	350 (10.8)		372 (30.8)	104 (13.2)		493 (28.4)	137 (10.4)		100 (13.6)	24 (4.5)	
No response	37	36		9	11		15	11		22	5	
Alcohol consumption												
Non-drinker	1176 (38.2)	1612 (49.5)	**<0.001**	364 (30.0)	290 (36.8)	**0.002**	661 (38.0)	656 (49.6)	**<0.001**	440 (59.8)	377 (70.3)	**<0.001**
Drinker	1899 (61.8)	1646 (50.5)		848 (70.0)	498 (63.2)		1078 (62.0)	666 (50.4)		296 (40.2)	159 (29.7)	
No response	29	32		4	9		15	10		20	3	
Physical activity												
No	1666 (56.7)	1670 (52.9)	**<0.001**	536 (46.1)	341 (44.3)	0.441	1006 (60.1)	638 (50.2)	**<0.001**	491 (70.1)	324 (62.1)	**0.003**
Yes	1273 (43.3)	1489 (47.1)		626 (53.9)	428 (55.7)		667 (39.9)	634 (49.8)		209 (29.9)	198 (37.9)	
No response	165	131		54	28		81	60		56	17	
BMI, kg/m^2^	24.15 ± 3.75	23.69 ± 3.32	**<0.001**	23.83 ± 4.19	22.92 ± 3.69	**<0.001**	24.44 ± 3.42	23.65 ± 3.13	**<0.001**	24.19 ± 3.26	24.09 ± 3.09	0.593
WC, cm	83.29 ± 10.59	81.13 ± 9.75	**<0.001**	80.76 ± 11.70	77.49 ± 10.61	**<0.001**	83.95 ± 9.47	80.60 ± 8.95	**<0.001**	86.09 ± 8.90	85.03 ± 9.02	**0.036**
WHtR	0.50 ± 0.07	0.50 ± 0.06	0.426	0.48 ± 0.06	0.47 ± 0.06	**<0.001**	0.51 ± 0.06	0.50 ± 0.05	**<0.001**	0.54 ± 0.06	0.54 ± 0.06	0.597
ABSI	77.65 ± 4.7	77.47 ± 4.58	0.139	75.09 ± 3.87	74.71 ± 4.08	**0.037**	77.87 ± 3.88	77.14 ± 3.95	**<0.001**	81.83 ± 4.30	81.34 ± 4.16	**0.041**
BRI	3.51 ± 1.26	3.47 ± 1.18	0.283	3.01 ± 1.18	2.84 ± 1.06	**<0.001**	3.62 ± 1.09	3.41 ± 1.00	**<0.001**	4.26 ± 1.27	4.22 ± 1.27	0.597
hs-CRP, mg/L	1.24 ± 2.05	1.11 ± 1.95	**0.013**	1.13 ± 1.92	1.04 ± 2.03	0.287	1.17 ± 1.76	0.98 ± 1.61	**0.003**	1.54 ± 2.70	1.41 ± 2.44	0.398

Data were presented as mean ± standard deviation or number (percentage). χ^2^ test and Student’s *t* test were conducted for categorical and continuous variables, respectively. Bold numbers highlight the statistical significance. Abbreviations: ABSI, a body shape index; BMI, body mass index; BRI, body roundness index; hs-CRP, high-sensitivity C-reactive protein; SD, standard deviation; WC, waist circumference; WHtR, waist-to-height ratio.

## Data Availability

The data are freely available on the website (https://knhanes.kdca.go.kr/knhanes/main.do (accessed on 12 August 2022)).

## Supplementary Materials

The following supporting information can be downloaded at: https://www.mdpi.com/article/10.3390/nu14173461/s1, Figure S1: The scatter plot between log transform of serum folate and body mass index.
