# Supplementary material for: Serum Folate Related to Five Measurements of Obesity and High-Sensitivity C-Reactive Protein in Korean Adults
Source: Nutrients. 2022 Aug 24;14(17):3461. doi: 10.3390/nu14173461 (PMC9459859; doi:10.3390/nu14173461)
Supplement: Supplementary file 1 [file nutrients-14-03461-s001.zip › nutrients-1837629-supplementary.pdf]

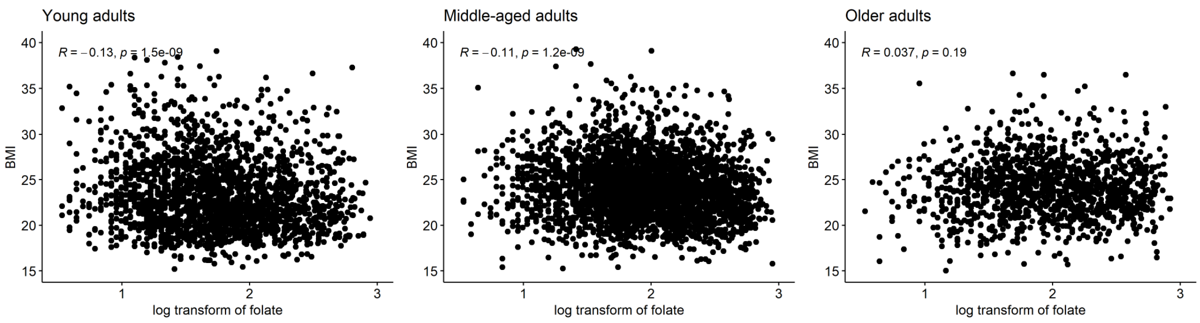

Supplementary Figure S1.The scatter plot between log transform of serum folate and body mass index.
